# A Clean Milk Opportunity

**Published:** 1923-02

**Authors:** 


### A CLEAN-MILK OPPORTUNITY.

### EFFECTS OF THE NEW REGULATIONS.

THE beginning of the year finds England one step further on the road to becoming a clean-milk country. Henceforward it is possible for milk to be delivered to the general public under official licence as to its bacterial content and to be labelled, according to its purity, "Certified," "Grade A (Tuberculin Tested)," "Grade A," or "Pasteurised." Such grading under licence is not, of course, entirely new, for during the last year or so it has been possible to obtain official recognition as a producer of clean "graded" milks, but the matter is now placed upon a far more satisfactory basis by making it incumbent upon local authorities themselves to inspect, and to issue licences to those dairymen who apply for them if their milk is consistently satisfactory. Once the licence is granted, periodical milk analyses will insure the maintenance of the required standard.

### The Weak Point.

On the face of it all this sounds hygienically excellent, but as far as achievement of a national supply of clean milk is concerned there is still one very weak point. Dairymen are in no way legally bound to hold these licences. If the public is foolish enough still to accept heavily infected milk, it will be delivered to them indefinitely. The local health authority cannot compel the production of clean milk, and as long as licensing is optional for the producers, the effectiveness of the new regulations must depend entirely upon a widespread determination to deal with licence-holders only. Another point of danger lies in the fact that the licensed firms will probably find in their cleanliness an excuse for increased prices, and it would seem that the only certain way of preventing this is to make licensing compulsory. Possibly compulsion will be the next stage in the clean-milk campaign, but meanwhile our only defence against prices impossible for the poor (the class to which clean milk is of vital importance) will be a public boycott of the unlicensed dealers.

### Bacteria, Strictly Limited.

How far this can be achieved depends in great measure upon public enlightenment. Several excellent bodies—chief, perhaps, the National Clean Milk Society—have long devoted themselves to this difficult task, and, in view of their many publications, it is needless here to dwell upon the manifold horrors of really dirty milk. The reforms which ought to be made—the adoption of clean depots, proper bottling plants, cooled milk-vans, &c.—are as well known as they are difficult separately to enforce, and therefore licensing of milk as delivered has one paramount advantage. To keep the number of bacteria present at the time of delivery down to the limits prescribed in his licence, the dairyman and all his colleagues must have handled, covered and cooled the milk correctly. If they do not achieve all these things at the outset, they will not gain their licence, because by other means they cannot give us clean milk; if they subsequently fail to maintain these standards, the licence will, after warning, be withdrawn.

